# Mapping Social Vulnerability and Proximity to Pharmacies in Minnesota, 2009–2024

**DOI:** 10.5888/pcd23.250451

**Published:** 2026-07-09

**Authors:** Emma Goldner, Emily Styles, Lindsay Sorge, Anjoli Punjabi, Olihe Okoro, Sarah Westberg, Chrystian Pereira, James Peacock

**Affiliations:** 1 Minnesota Department of Health, St. Paul, Minnesota; 2 University of Minnesota College of Pharmacy, Minneapolis, Minnesota; 3 University of Minnesota College of Pharmacy, Duluth, Minnesota

**Figure:**
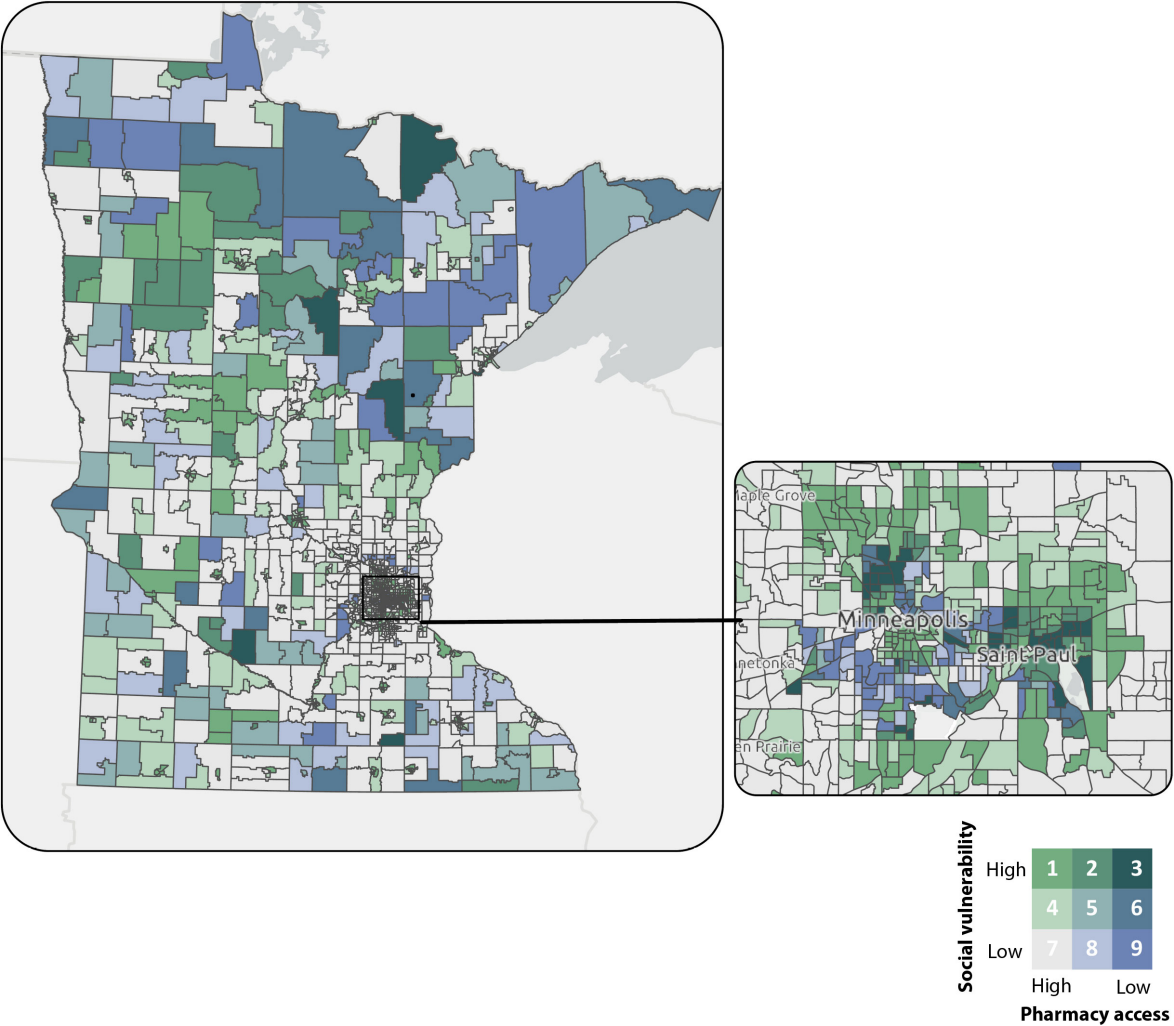
Bivariate map of Social Vulnerability Index (SVI) (categorized as low, moderate, and high) and pharmacy access (categorized as low, limited, or high) in Minnesota, 2024. SVI values range from 0 to 1, with higher scores indicating greater social vulnerability. Numerals in the key indicate the following: 1 = high SVI, high access; 2 = high SVI , limited access; 3 = high SVI, low access; 4 = moderate SVI, high access; 5 = moderate SVI, limited access; 6 = moderate SVI, low access; 7 = low SVI, high access; 8 = low SVI, limited access; 9 = low SVI, low access. Data sources: Centers for Disease Control and Prevention (1), US Census Bureau (2), Minnesota Board of Pharmacy (3).

## Purpose

From 2009 to 2024, Minnesota lost 13% of its community pharmacies. Pharmacy closures can increase barriers to preventive care. Pharmacies are a source of reputable medical advice and support for patient self-management and other clinical services and are an access point for vaccinations and medications. Without these services, patients may not receive or take their medication on time, which may worsen chronic conditions, increase the number of hospitalizations, and result in poorer health.

Community health inequities can be exacerbated by barriers to accessing care, such as distance to a pharmacy. Pharmacy deserts are typically defined as areas where residents have difficulty accessing a pharmacy for economic reasons or because of distance. A national study describing the relationship between social vulnerability and pharmacy access did not provide a detailed picture of access in Minnesota (4). Our analysis aimed to understand how proximity to a pharmacy intersects with social vulnerability at 3 geographic levels and expand beyond indicators typically included in definitions of pharmacy deserts. Our analysis is a first step in refining a definition of pharmacy deserts for communities in Minnesota.

## Data and Methods

We used Minnesota Board of Pharmacy lists of active licensed Minnesota pharmacies from 5 time points from 2009 through 2024 (3). We used ArcGIS Pro 3.3.0 (Esri) to geocode publicly accessible pharmacies and Network Analyst tools to measure driving distance between the closest 2 pharmacies and the population center of each census tract.

Distance thresholds used in previous studies of barriers to pharmacy access have varied; however, many used a threshold of 1 mile in urban areas (5,7). Analysis of rural areas has been less common, and thresholds have varied from 10 to 15 miles (7–9). Based on its review of the literature, the study team decided that 3 tiers would fit best for Minnesota: urban core, metropolitan, and nonmetropolitan.

We classified census tracts into 3 categories of pharmacy access: low access, limited access, and high access. We defined low access as a distance to the closest pharmacy of more than 1 mile in the Minneapolis–St. Paul urban core, more than 5 miles in metropolitan areas, and more than 15 miles in nonmetropolitan areas. Limited-access tracts were defined as having only 1 pharmacy within the distance threshold; these would become low-access tracts if the closest pharmacy were to close. High-access tracts were defined as those served by 2 or more pharmacies.

We used the urban core designation from the Metropolitan Council (10), a policy making and planning agency for the 7-county Minneapolis–St. Paul metropolitan area. It describes communities that were planned and largely developed with a grid street pattern that includes consistent sidewalks and mixed-used development with residential and commercial areas in close proximity. The urban core has slower speed limits, more frequent stops, and less frequent on-site parking. Driving 1 mile in the urban core generally takes longer than driving 1 mile in rural and other metropolitan communities.

We used the 2022 Social Vulnerability Index (SVI) (1) to identify social vulnerability in each census tract. SVI scores describe demographic and community characteristics across 4 themes: socioeconomic status, household characteristics, racial and ethnic minority status, and housing type and transportation. SVI scores range from 0 to 1, with higher scores indicating greater social vulnerability. We defined high vulnerability as a score of 0.75 or higher (fourth quartile), moderate vulnerability as a score of 0.5 to less than 0.75 (third quartile), and low vulnerability as a score of 0 to less than 0.5 (first and second quartile). We used 2018–2022 American Community Survey 5-year estimates from the US Census Bureau to assess population sizes (2).

### Statistical analysis

We compared the median SVI score among low-access, limited-access, and high-access tracts and used the Kruskal–Wallis test to detect significant differences among them. In further analysis, we compared the median SVI score between low-access tracts existing in 2009 and low-access tracts that had developed by 2024.

## Highlights

Of 1,498 populated census tracts in Minnesota, 147 had low pharmacy access and 207 had limited pharmacy access (Table), representing 1,130,177 people in Minnesota, or 19.9% of the total population. The urban core had the largest percentage of people living in a low-access or limited-access tract: 63.2% of the population, or 580,992 people. Nonmetropolitan areas were next, with 19.2% of the population (420,655 people) in a low-access or limited-access tract. Low-access and limited-access tracts had higher median SVI scores than high-access tracts in Minnesota overall (0.534 and 0.569 vs 0.473, respectively; *P* < .001) and in nonmetropolitan areas (0.507 and 0.563 vs 0.488; *P* = .007). In the urban core, we found no significant differences in median SVI across pharmacy access levels. In metropolitan tracts, we found that low-access and limited-access tracts had lower SVI scores than high-access tracts (0.068 and 0.241 vs 0.422; *P* <.001). Some of these low-access areas coincided with affluent neighborhoods, where residents have resources to access services despite living farther away.

**Table:** Median SVI^a^ Scores of Populated Census Tracts in Minnesota, by Type of Census Tract and Access to Pharmacies,^b^ 2024^c^

Type of census tract	No. of census tracts	Median SVI score			*P* value^d^
		Low access (147 tracts)	Limited access (207 tracts)	High access (1,144 tracts)	
All	1,498	0.534	0.569	0.473	<.001
Urban core	258	0.663	0.770	0.738	.16
Metropolitan	603	0.068	0.241	0.422	<.001
Nonmetropolitan	637	0.507	0.563	0.488	.007

Abbreviation: SVI, Social Vulnerability Index.

a The 2022 SVI was used to identify the vulnerability level for each census tract (1). The SVI ranges from 0 to 1, with higher scores indicating greater social vulnerability. A score ≥0.75 was considered a high level of social vulnerability.

b Low access defined as a distance to the closest pharmacy of >1 mile in the Minneapolis–St. Paul urban core, >5 miles in metropolitan areas, and >15 miles in nonmetropolitan areas. Limited-access tracts defined as having only 1 pharmacy within the distance threshold. High-access tracts defined as those served by ≥2 pharmacies.

c Data sources: Centers for Disease Control and Prevention (1), US Census Bureau (2), Minnesota Board of Pharmacy (3).

d Calculated by using Kruskal–Wallis test.

From 2009 to 2024, twenty-four tracts with 77,000 residents became low-access tracts because of pharmacy closures. The median SVI of these tracts was significantly higher than the median SVI of the low-access tracts that had existed as of 2009 (0.676 vs 0.473, *P* = .02).

## Action

Our analysis is the first examination of how social vulnerability and pharmacy access intersect in Minnesota. A strength of our study is that we chose distance thresholds customized to Minnesota after a literature review showed inconsistent thresholds in nonurban areas and lack of alignment with the various communities in Minnesota. Adapting definitions is important to align with the needs and characteristics of assessed communities. We found that areas of low pharmacy access were not necessarily more socially vulnerable; however, recent loss of access had occurred in socially vulnerable communities. This map highlighted areas in Minnesota that were most affected by lack and closure of pharmacies.

One limitation of the SVI at the census tract level is that it assumes that all residents in that tract experience the same level of social vulnerability. Residents who are socially disadvantaged may be overlooked in a census tract where they are not the majority. Additionally, rural Minnesota has many census tracts that have a small population and a pharmacy, yet many residents may still have low levels of access to a pharmacy because the calculated distance from that pharmacy may be more than 15 miles from the weighted population center for that tract.

Our results have guided the development of a definition of a pharmacy desert in Minnesota that includes the distance thresholds used in our study and incorporates components of the SVI. Policy changes to support community pharmacies are needed to address areas of low access and maintain access where it is limited. Our analysis sheds light on the problem of barriers to pharmacy access and provides critical data to policymakers. Previous iterations of maps from this work have been used to support policy initiatives enacted in the Minnesota legislature.
